# Multiorgan Failure in Hemophagocytic Lymphohistiocytosis Secondary to Acute Monocytic Leukemia and Myocarditis in a Pediatric Patient

**DOI:** 10.7759/cureus.39147

**Published:** 2023-05-17

**Authors:** Scott Everett, Alice M Dalo, Andrew L Alejo, Joseph Ptasinski

**Affiliations:** 1 College of Medicine, Northeast Ohio Medical University, Rootstown, USA; 2 Pediatrics, Akron Children's Hospital, Akron, USA

**Keywords:** pediatrics, inflammatory cardiomyopathy, hemophagocytic lymphohistiocytosis, acute monocytic leukemia, myocarditis

## Abstract

Acute monocytic leukemia (AML), a subtype of acute myeloid leukemia, is a rare leukemia found in children. It occurs more frequently in adults over the age of 60. Myocarditis represents inflammation of the muscular layer of the heart, the myocardium causing weakening of the muscles that can lead to hemodynamic instability from a reduced ejection fraction. Myocarditis in the pediatric population is most commonly secondary to a viral or infectious etiology. Hemophagocytic lymphohistiocytosis (HLH) is a rare condition of immune dysregulation characterized by severe organ damage induced by an increased inflammatory response and uncontrolled T-cell and macrophage activation. In this case report, we examine a rare presentation of leukemic myocarditis in the presence of HLH, which displays an uncommon cause of an inflammatory state with several complicated concomitant diagnoses. Our patient developed severe multiorgan dysfunction involving liver and kidney failure that required prolonged critical care support, and the patient expired due to his multiorgan failure. We highlight the unusual clinical presentation of myocarditis in the setting of HLH and AML in this complicated pediatric patient and aim to improve outcomes of patients presenting similarly in the future.

## Introduction

Acute monocytic leukemia (AML), a subtype of acute myeloid leukemia, is a rare leukemia found in children, with acute lymphoblastic leukemia being the most common. AML is primarily found in adults; however, in children, it usually presents within the first two years of life or during the teenage years. In the United States, only 730 people under the age of 20 years old are diagnosed with AML each year [1]. The five-year survival rate for children under the age of 15 with AML is 68%, which decreases to 67% in teens ages 15 to 19 [2]. AML is associated with complications such as infection, tumor lysis syndrome, disseminated intravascular coagulation, myocarditis, and hemophagocytic lymphohistiocytosis (HLH) [2].

HLH is a rare condition of immune dysregulation, that can be primary or secondary, and is characterized by severe organ damage induced by an increased inflammatory response and uncontrolled T-cell and macrophage activation [3]. This damage can be observed in the bone marrow, central nervous system, and liver [4]. The increased inflammatory response of the immune system causes an increase in histiocytes and lymphocytes to accumulate in organs causing localized damage. HLH most commonly affects infants and young children and can be inherited or be secondary to an infection. Familial HLH usually affects children under the age of one, while the secondary form typically occurs after age six. Symptoms include fever, cytopenias, hepatosplenomegaly, abdominal distension, failure to thrive, rash, jaundice, malaise, and enlarged lymph nodes. Diagnostic criteria must include five of eight following parameters: fever, splenomegaly, hypertriglyceridemia and/or hypofibrinogenemia, hyperferritinemia, low or absent natural killer cell activity, hemophagocytosis, cytopenias, or elevated soluble interleukin-2 receptor (CD 25). In this case, we describe a 13-year-old boy who was diagnosed with myocarditis and multisystem inflammatory syndrome in children (MIS-C) in the setting of both AML and HLH.

## Case presentation

**Figure 1:**
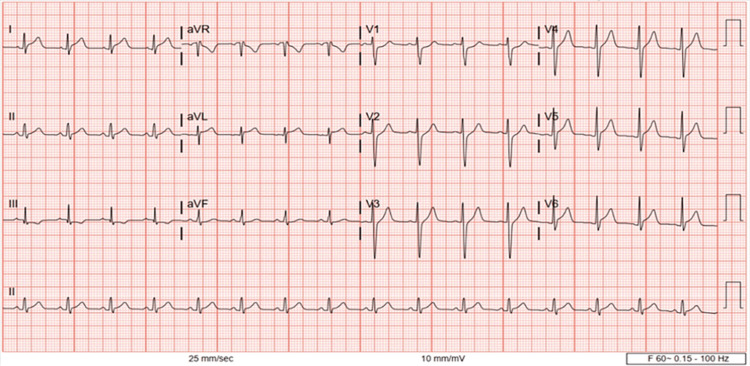
Electrocardiogram on day one which showed sinus rhythm with a normal QRS vector, and RSR’ in aVR (likely a normal variation)

**Figure 2:**
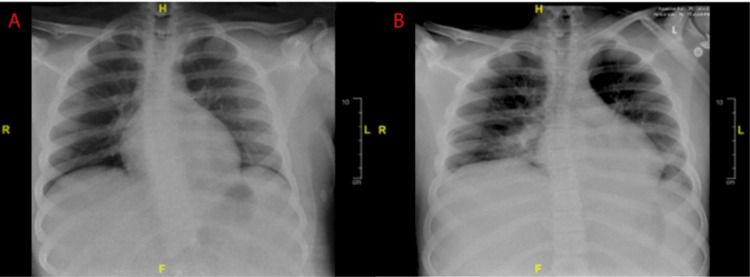
Chest x-ray Chest x-ray taken on day one displays symmetric lung aeration with mildly low lung volumes and crowding of central markings with no airspace disease or pleural disease abnormality identified (A). Chest x-ray taken on day six displays lower lung volumes with slightly increased bibasilar opacities. Left greater than right with partial obscuration of the left hemidiaphragm (B).

Our patient was a 13-year-old Caucasian male with a past medical history of attention deficit hyperactive disorder, Tourette's, and asthma who presented to the emergency department with three to four days of persistent upper respiratory tract infection symptoms, intermittent fevers ranging from 99°F to 100.6°F, positional chest and neck pain, palpitations, and dyspnea. He previously presented to another emergency department (ED) a week prior with similar symptoms with elevated troponins and splenomegaly and was diagnosed with myocarditis. His symptoms persisted so he arrived at our ED for additional investigation. Initial laboratory results displayed significantly elevated troponins of 820 ng/L and a C-reactive protein (CRP) of 58. An electrocardiogram showed ST elevations in aVL with nonspecific depressions anteriorly (Figure 1). His complete blood count (CBC) was indicative of mild anemia and 12% blast cells in the peripheral smear. Chest x-ray (CXR) at the time was nonsignificant; however, on day six, it showed bibasilar diffuse opacities (Figures 2A, 2B). A respiratory viral panel, *Toxoplasma*, *Bartonella*, Lyme, and parvovirus B19 serologies were all negative at this time. The patient’s clinical picture was concerning for myocarditis of viral etiology versus pericarditis so he was transferred to the pediatric intensive care unit (PICU) for further evaluation.

Upon admission to the PICU, the patient was started on high-dose solumedrol and insulin for possible multisystem inflammatory syndrome in children (MIS-C) and hyperglycemia. He was placed on 1 L O_2_ via nasal cannula for comfort. An echocardiogram at this time demonstrated a mildly depressed left ventricular ejection fraction of 45%. He was started on milrinone due to his low ejection fracture but was discontinued shortly due to hypotension and then started on epinephrine for three days for his hemodynamic lability. He also received two doses of intravenous (IV) furosemide 10 mg with intravenous immunoglobulin (IVIG) for diuresis and hypertension which improved his blood pressure.

On day three, a repeat echocardiogram showed a decrease in the ejection fraction to 27%; however, his condition was stable so the decision was made to transfer him to the floor. B-type natriuretic peptide (BNP), CRP, and troponins were all downtrending; however, on physical examination, the patient could not stand or walk for more than 15-30 seconds due to nausea, abdominal pain, and dizziness. At this time, hematology was consulted due to significant leukocytosis of 61,000 with monocytosis. They determined there was low suspicion for an oncological process based on his clinical picture in combination with his labs; however, they did note his monocytosis was most likely reactive from a probable infection. The next day, the patient’s oxygen requirement increased to 2 L as he was dyspneic. A repeat CBC was significant for a white blood count of 149,200 and thrombocytopenia at 39,000 (predominance of monocytes), concerning for malignancy (Table 1). His follow-up echocardiogram showed mild to moderate depressed LV systolic function with an ejection fraction of 45% with preservation of right ventricular function. After discussion with oncology, cardiology, and the PICU team, it was recommended that the patient be transferred back to the PICU due to suspected malignancy and return of depressed systolic function.

**Table 1:** Laboratory values

	Day 1	Day 4	Day 7	Reference
Hemoglobin	12.9	9.4	8.2	13-15.2 g/dL
White blood cells	6,200	149,200	127,800	4,800-10,800/mm^3^
Troponin T	820	1,164	394	0-14 ng/L
NT pro-B-type natriuretic peptide	1,238	1,917	1,759	<178 pg/mL
Creatinine	0.78	1.23	2.18	0.50-0.80 mg/L
C-reactive protein	6.8	2.1	1.4	0.0-1.0 mg/dL

Upon return to the PICU on day five, a bone marrow biopsy was performed and showed findings for AML. At this time, the patient needed an increasing oxygen requirement and was started on a high-flow nasal cannula. A CXR was taken which showed low lung volumes so the decision was made to escalate to bilevel airway pressure (BiPAP) due to his worsening hypoxia. The fraction of inspired oxygen (FiO_2_) remained high and worsening oxygen saturation required intubation the next day and placement on a bilevel positive airway pressure machine. Shortly after intubation, he became acutely hypotensive despite volume resuscitation so a bumetanide bolus/infusion was ordered as well as increased vasopressor support. He went into two cardiopulmonary arrests with a return of spontaneous circulation which prompted placement on venoarterial extracorporeal membrane oxygenation (VA-ECMO). A repeat CXR displayed lower lung volumes with slightly increased bibasilar opacities and left greater than right obscuration of the left hemidiaphragm (Figure 2). On day seven, the patient was febrile with a ferritin of 41,252, hemoglobin of 8.2, hematocrit of 26.1, a fibrinogen of 125, and a white blood count of 127.8 and was diagnosed with hemophagocytic lymphohistiocytosis, given his fulfillment of five of eight required parameters. The patient continued to have distributive shock requiring significant inotropic support.

On day eight, the patient remained vasoplegic and hypotensive on VA-ECMO despite high doses of epinephrine, norepinephrine, and vasopressin. The decision to start levothyroxine, methylene blue, hydrocortisone infusions, hydroxocobalamin, thiamine, and vitamin C was made; however, the patient showed no improvement. He started to have active bleeding from his endotracheal tube with increasing intra-abdominal pressures prompting a computed tomography (CT) abdomen to be ordered. He also developed increasing transaminitis, worsening coagulopathy, and persistent renal failure on labs. Due to his active bone marrow failure based on his decreasing hemoglobin, he received multiple blood product transfusions with no improvement and a WBC <100 with leukoreduction and chemotherapy. For the first time on this visit, upon examination of his eyes, his right pupil was 5 mm and non-reactive with pupil asymmetry. An electroencephalogram was ordered and showed suspicion of right cerebral dysfunction that was unable to be confirmed with a CT scan due to the patient’s hemodynamically unstable condition. His ammonia levels were in the 150s, with signs of hepatic encephalopathy on physical examination.

Due to his refractory multisystem organ failure, the family was informed there were no medical, surgical, or mechanical support options that would offer a meaningful opportunity for survival. Extracorporeal life support, inotropic, and ventilatory support were discontinued. The patient expired soon after the withdrawal of care and making him comfortable with appropriate medications.

## Discussion

We report a unique case of both myocarditis and HLH in a patient with AML. His presentation was unusual, and he had a guarded prognosis. The lack of reported cases of AML with unique presentations in children has contributed to the challenges in making a prompt diagnosis. HLH, in particular, is diagnosed clinically but usually is a diagnosis of exclusion based on the patient’s symptoms. Criteria for diagnosing HLH include fever ≥38.5°C, splenomegaly, peripheral blood cytopenia, with at least two of the following: hemoglobin <9 g/dL, platelets <100,000/μL, absolute neutrophil count <1,000/μL, hypertriglyceridemia (fasting triglycerides >265 mg/dL) and/or hypofibrinogenemia (fibrinogen <150 mg/dL), hemophagocytosis in the bone marrow, spleen, lymph node, or liver, low or absent NK cell activity, or ferritin >500 ng/mL [5]. Many of these criteria can be attributed to other pathologies, so more often the more common diseases are ruled out first, leading to a delay in an HLH diagnosis. Prompt dismissal of usual pathologies would allow clinicians to diagnose HLH quicker, prompting initiation of etoposide, intrathecal methotrexate, and dexamethasone sooner. These medications have been shown to help decrease the inflammation shown in HLH, providing a better outcome for the patient [3].

Our patient presented with multiple complaints, and throughout his hospital stay, his health was rapidly declining while his whole healthcare team implored the most severe countermeasures in order to treat him based on his various symptoms and new laboratory and radiographic findings daily. His myocarditis in addition to his respiratory failure promoted multiple cardiopulmonary arrests. His AML, shown on his labs, put him at an increased risk of immunosuppression which allowed his immune system to be dysregulated attributing to his HLH diagnosis which was found on day seven out of eight in his hospital stay. HLH should be suspected in a patient with fever and multiorgan involvement, which cannot be attributed to any other apparent cause. Overall, there were multiple factors that this patient was being monitored and promptly treated for; unfortunately, there were no additional measures available for this patient given his complex situation.

Few case reports have been presented that show HLH, AML, and additional comorbidities in pediatric patients. A recent case report on HLH by Alsaid et al. displayed a child with relapsing polychondritis while also being diagnosed with acute myelogenous leukemia [6]. This patient presented with fever, costochondritis, splenomegaly, thrombocytopenia, and anemia and was treated similarly to our patient with steroids, given that steroids have been shown to be effective in HLH with known solid tumor malignancy. Their hospital stay resulted in multiorgan failure as well, leading to them to succumb to their illness. Delavigne et al. reported on 32 patients developing HLH during treatment with chemotherapy for acute myeloid leukemia; however, the median age of patients was 59 years old with no pediatric patients [7]. These patients had symptoms such as fever, hepatosplenomegaly, icterus, rash, and respiratory distress, similar to our patient.

AML is a complex disease with a variety of presentations. It appears that patients with AML may be more prone to developing HLH, as well as additional diseases that can collectively negatively affect the patient’s overall health status. Being able to recognize that AML may present in conjunction with other rare diseases is important in diagnosing and treating the patient swiftly. Although the outcomes of these patients are grim, full supportive measures and medical intervention are necessary to ensure quality patient care.

## Conclusions

To our knowledge, this is the first time a pediatric patient was diagnosed with myocarditis and HLH in the setting of AML. HLH alone has a poor prognosis, and in the presence of increased immunosuppression due to AML, being able to successfully treat the patient becomes increasingly difficult. All interventions were untaken to help this patient try and recover from the multitude of diseases he was experiencing, even using VA-ECMO after two cardiopulmonary arrests. The patient experienced a rapid decline in health over a brief course in the hospital. This case widens the spectrum of AML complications and may provide perspective on diagnosis and treatment for clinicians faced with similar patients.
